# 

**Published:** 1851-04

**Authors:** 


### CONTENTS.

### JOURNAL.

Article I.—Treatment of Dental Caries, complicated with Affections of the Pulp and Peridental Membrane. By Robert Arthur, D. D. S.... 229
Article II.—Cheap Artificial Teeth, and Cheap Dental Operations in England,.... 240
Article III.—A New Kind of Artificial Palate: with Observations on the Changes that take place in the Velum and Pharynx, during Deglutition, Respiration and Articulation. By Dr. S. P. Hullihen, of Wheeling, Va.... 245
Article IV.—Daily Jottings in the Gold Book of a Dentist—January 1, to March 1. By E. Townsend, D. D. S.—No. 1,.... 252
Article V.—A Test of Levett's Patent Enamel, and its Discovery. By C. T. Cushman, D. D. S., Columbus, Geo.... 263
Article VI.—Observations on Exostosis. By D. Vandenburgh,.. 268

### SELECTED ARTICLES.

Article VII.—James Taylor, M. D., on Filling Teeth,.... 273
Article VIII.—Treatment of Dental Pulp, preparatory to Plugging. By J. D. White, Dentist,.... 297
Article IX.—Mechanical Dentistry. By T. L. Buckingham, D. D. S., Philadelphia,.... 305
Article X.—Atmospheric Pressure. By James Flemming, M. D., Harrisburg, Pa.... 308
Article XI.—A Case of Reproduction of the Inferior Maxilla after Necrosis from Phosphorus Vapor,.... 311
Article XII.—Cancerous Tumor within the Antrum—Removal of Superior Maxillary Bone under the Influence of Chloroform, 312
Article XIII.—Fatal Results from the Inhalation of Chloroform, 315
Article XIV.—Two Cases in which the Hair, Teeth and Skin were very Imperfectly Developed,.... 320
Article XV.—Fistula Lachrymalis caused by Syphilis,.... 321
Article XVI.—Medicine and Surgery in Egypt,.... 322
Article XVII.—A Lecture on the Diseases of the Inferior Maxilla, Delivered by John Simon, Esq., Surgeon to St. Thomas' Hospital,.... 332
Article XVIII.—Microscopical Researches of Dr. Th. Schwann into the Structure of the Teeth,.... 348
Article XIX.—Two Cases of Successful Removal of the Upper Jaw, on account of Malignant Disease of the Antrum. By Robert M. Macdonnell, M. D., &c., of Montreal,.... 362
Article XX.—Periostitis of the Upper Jaw, in connection with the Habit of Biting Coin,.... 369
Article XXI.—On the Appearance of the Gums in Phthisis,.... 372
Article XXII.—Displacement of the Epiglottis. By Jas. Kitsell, Esq.... 374

### BIOGRAPHICAL DEPARTMENT.

Biographical Notice of the late Dr. James Gardette, Surgeon Dentist, of Philadelphia. By Emile B. Gardette, M. D., Dentist,.... 375

### BIBLIOGRAPHICAL DEPARTMENT.

A Much Less Painful and More Scientific Method of Extracting Teeth. By H. Gilbert, M. R. C. S. L., &c., &c.......... 382
On Tic Douloureux and other Painful Affections of the Nerves, with Suggestions for their Treatment by means of the Aneuralgicon. By Toogood Downing, M. D........................... 386
A few Practical Observations on Diet, especially that of Children and Invalids, with Inquiry into the effects of Alcohol on the System. By Charles Beckett, M R. C. S................ 386
A Practical Treatise on Dental Medicine: being a Compendium of Medical Science as connected with the Study of Dental Surgery. By Thos E. Bond, A. M., M. D., &c., &c.......... 387
The Medical Student's Guide in Extracting Teeth: with numerous Cases in the Surgical Branch of Dentistry. With Illustrations. By S. Hornor, Practical Dentist,............. .......... 388
Review of Chemistry for Students, adapted to the Courses as taught in the Principal Medical Schools of the United States. By Jno. G. Murphy, M. D................................ 390
Ether and Chloroform; their Employment in Surgery, Dentistry, Midwifery, Therapeutics, &c. By J. F. B. Flagg, M. D., Surgeon Dentist, Member of the Rhode Island Med. Society, 390
An Inaugural Essay on Zoo-Adymia. By Geo. J. Zeigler, M. D... 390
A Treatise on the Diseases and Surgical Operations of the Mouth and Parts Adjacent: with Notes of Interesting Cases, Ancient and Modern. By M. Jourdain, Dentist, &c............... 391
A Systematic Treatise, Historical, Etiological and Practical, on the Principal Diseases of the Interior Valley of North America, as they appear in the Caucasian, African, Indian and Esquimaux Varieties of its Population. By Daniel Drake, M. D...... 392
An Introductory Lecture, delivered at the Opening of the Thirty-first Session of the Medical College of Ohio. By John Bell, M. D., Professor of the Theory and Practice of Medicine, &c....... 393
Success in the Medical Profession: An Introductory Lecture, delivered at the Mass. Med. College. By John Ware, M. D., Hersey Professor of the Theory and Practice of Medicine in Harvard University,.......................................... 393
Maryland Hospital for the Insane—Reports for the Year 1850,..... 394
Pennsylvania Hospital Report for the Year 1850,................ 394
Proceedings of the American Philosophical Society, April, Dec., 1850, 394

### EDITORIAL DEPARTMENT.

Dr. Gardette on the Propriety of Abolishing Dental Colleges, and Substituting for them a Lectureship on Dentistry in each of the Medical Schools,.................................... 395
Acidulated Tooth Powders,............................. ........ 401
Ulceration of the Tongue Cured by the Removal of Two Amalgam Fillings from the Teeth,.................................. 402
Stockton's New Style of Mineral Teeth,......................... 403
Annual Commencement of the Baltimore College of Dental Surgery, 403
Corundum Wheels,............................................. 404
John D. Chevalier,............................................ 404
The Journal,................................................. 404

